# A16 UNMET HEALTHCARE NEEDS AMONG PEOPLE WITH INFLAMMATORY BOWEL DISEASE: A NATIONAL CROSS-SECTIONAL POPULATION-REPRESENTATIVE STUDY

**DOI:** 10.1093/jcag/gwac036.016

**Published:** 2023-03-07

**Authors:** G Postill, E I Benchimol, J Im, A Tang, E Kuenzig

**Affiliations:** 1 Temerty Faculty of Medicine, University of Toronto; 2 SickKids Inflammatory Bowel Disease Centre, Division of Gastroenterology, Hepatology and Nutrition, Department of Paediatrics, The Hospital for Sick Children; 3 Child Health Evaluative Sciences, SickKids Research Institute; 4 Institute of Health Policy, Management and Evaluation, University of Toronto; 5 Institute for Clinical and Evaluative Sciences, Toronto, Canada

## Abstract

**Background:**

Inflammatory bowel disease (IBD) is a chronic inflammatory disease primarily affecting the gastrointestinal tract. Despite treatment with the current standards of care, many IBD patients experience relapsing, remitting, and disabling bowel symptoms and significant disease complications. Ensuring patients have adequate access to high quality multidisciplinary healthcare is vital for the short- and long-term wellbeing of IBD patients.

**Purpose:**

(1) Compare unmet healthcare needs of people with and without IBD. (2) Determine whether accessing regular medical care mediates the association between IBD and unmet healthcare needs. (3) Describe the reasons for unmet healthcare needs among people with and without IBD.

**Method:**

We used the 2014 Canadian Community Health Survey, a population-representative national cross-sectional survey conducted by Statistics Canada. Respondents with a non-IBD bowel disorder or aged 18 or younger were excluded. Survey weights were used for descriptive statistics. We used multilevel logistic regression to compare perceived unmet healthcare needs among individuals with and without IBD, clustering by health region and controlling for age, immigration status, race, home ownership, marital status, annual household income, education level, and number of chronic conditions (0, 1, 2+). In a second model, we additionally controlled for having a regular family doctor, having consulted a specialist, and having consulted a psychologist to assess if regularly accessing medical care mediated the association between IBD and unmet healthcare needs. Individuals reporting unmet healthcare needs were asked about the reasons for their unmet healthcare needs. Responses are summarized with weighted percentages, plotted in a bar graph.

**Result(s):**

Among the 690 IBD and 62,832 non-IBD eligible survey respondents, 16.7% of people with IBD had an unmet healthcare need within the past 12 months, compared with 10.3% of those without IBD (OR 1.39, 95% CI 1.12 to 1.74). Additionally adjusting for regular access to medical care slightly attenuated the association between IBD and unmet healthcare needs (OR 1.29, 95% CI 1.03 to 1.62). Reasons for unmet healthcare needs differed among those with and without IBD (Figure). Specifically, doctors believing that care was unnecessary and the cost of care were more likely to be the reason people with IBD had an unmet healthcare need.

**Image:**

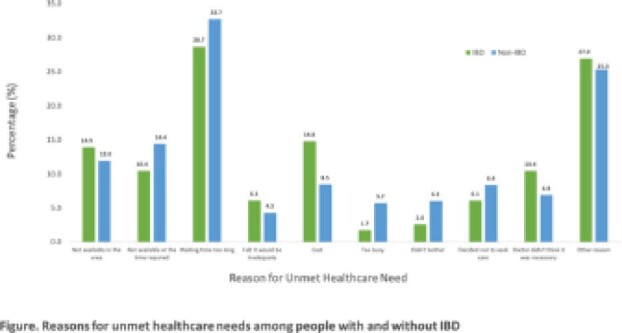

**Conclusion(s):**

People with IBD are more likely to have an unmet healthcare need that was partially mediated by access to healthcare professionals. Our work highlights the need for further research into the types of unmet healthcare needs experienced by people living with IBD. This underscores the need for multidisciplinary healthcare teams to address the increased burden of unmet healthcare needs in the IBD population.

**Please acknowledge all funding agencies by checking the applicable boxes below:**

None

**Disclosure of Interest:**

G. Postill: None Declared, E. Benchimol Consultant of: Hoffman La-Roche Limited and Peabody & Arnold LLP for matters unrelated to medications used to treat inflammatory bowel disease. Dr. Benchimol has also acted as a consultant for McKesson Canada and the Dairy Farmers of Ontario for matters unrelated to medications used to treat inflammatory bowel disease., J. Im: None Declared, A. Tang: None Declared, E. Kuenzig: None Declared

